# Berberine and Metabolic Dysfunction‐Associated Steatotic Liver Disease: Hope or Hype?

**DOI:** 10.1002/prp2.70300

**Published:** 2026-07-15

**Authors:** Ruth Nona, Eric Kalo

**Affiliations:** ^1^ College of Medicine and Dentistry James Cook University Smithfield Queensland Australia

**Keywords:** berberine, diabetes, MASLD, media, weight loss

Berberine, an odorless isoquinoline alkaloid derived from the *Berberis* species, has recently gained significant public attention as a metabolic supplement. Its popularity has been amplified by non‐medical messaging primarily through social media, where it is often promoted as “Nature's Ozempic”‐ a potential alternative to glucagon‐like peptide‐1 (GLP‐1) receptor agonists. This framing is appealing, but it is also potentially misleading. In this commentary, we pose a sharper question: does the current evidence justify viewing berberine as a meaningful adjunct in metabolic dysfunction‐associated steatotic liver disease (MASLD), or has enthusiasm outpaced the data?

Berberine has biologic plausibility and produces small‐to‐moderate effects on glucose and lipid parameters, yet existing human studies are short in duration, heterogeneous in design, and driven largely by surrogate endpoints. The clinical signal is therefore best interpreted as promising but unproven. This gap between preclinical promise and clinical impact exemplifies the challenge of translating metabolic supplements into outcomes.

Most evidence suggests AMPK activation underlies berberine's effects. Activation of AMPK inhibits SREBP‐1c, reducing hepatic lipogenesis and also lowers hepatic gluconeogenesis enzymes via phosphoenolpyruvate carboxykinase (PEPCK), AMP‐activated protein kinase (AMPK), and glucose‐6‐phosphate (G6Pase). Additional effects on LDL receptor expression and inflammation (NF‐κB) have been reported, but these are primarily from animal studies; upregulation of low‐density lipoprotein (LDL) receptors [1]. Other proposed mechanisms, including effects on SIRT1 and modulation of gut microbiota composition, remain biologically plausible but are supported mainly by preclinical studies. These pathways provide a mechanistic basis for observed metabolic effects; however, they do not necessarily translate into meaningful therapeutic effect. Importantly, human trials have focused on systemic outcomes (glycemia, lipids) rather than direct mechanistic biomarkers.

Berberine demonstrates modest, evidence‐based benefits in glycaemic and lipid control, but does not approximate the efficacy of established pharmacologic therapies such as that of statins [2]. Several, but relatively small, randomized trials show modest reductions in fasting glucose and HbA1c (typically 0.5%–1.0%). In addition, effects appear most consistent in early type 2 diabetes or insulin‐resistant states, with some studies suggesting modest glucose‐lowering effects; however, indirect comparisons with metformin are limited and do not support equivalence.

A typical HbA1c drop of < 1% is statistically significant but clinically less than standard drugs (metformin, GLP‐1RA). Furthermore, meta‐analyses demonstrate moderate reductions in LDL cholesterol and triglycerides, changes that are clinically relevant but remain inferior to statin therapy and should not be considered equivalent [3]. Evidence promoting berberine's role in weight loss remains inconclusive, with no rigorous scientific evidence to support this claim. Currently, reported reductions are small, ranging from 1 and 3 kg over weeks or months, and could be explained by enhanced metabolic signaling rather than appetite suppression. Claims that berberine mimics GLP‐1 agonism are largely unsupported. Collectively, the effects of berberine are real, but they are limited in magnitude and should not be overstated.

Metabolic dysfunction‐associated steatosis liver disease (MASLD), formerly called non‐alcoholic fatty liver disease (NAFLD), is now recognized as the leading cause of chronic liver disease worldwide, with significant associations with cardiometabolic and hepatic complications and mortality. In its initial stages, MASLD often presents without symptoms, but it can progress to cirrhosis and eventually hepatocellular carcinoma. Obesity and insulin resistance are key risk factors driving the onset and progression of MASLD. Consequently, weight reduction through dietary modification and increased physical activity remains the cornerstone of non‐pharmacological management for MASLD. In obese patients with both cirrhosis, even modest weight loss has been demonstrated to reduce portal pressure and may help avert decompensation [4]. Weight loss can further lead to improvement of liver transaminases, as well as reductions in liver fat, inflammatory markers, and consequently fibrosis [5, 6]. An ongoing phase 3, placebo‐controlled trial evaluating once‐weekly semaglutide at 2.4 mg demonstrated combined resolution of steatohepatitis and improvement in liver fibrosis in 32.7% of patients with MASLD receiving semaglutide, compared with 16.1% in the placebo group (estimated difference, 16.5 percentage points; 95% CI, 10.2–22.8; *p* < 0.001). (Funded by Novo Nordisk; ClinicalTrials.gov number, NCT04822181) [7].

In MASLD‐specific studies, the evidence remains even less convincing. Experimental studies suggest that berberine may help prevent steatosis from progressing to steatohepatitis and fibrosis by modulating lipid metabolism. It is believed to reduce de novo lipogenesis mediated by noticeably small to modest suppression by key enzymes such acetyl‐CoA (ACC), stearyl‐coenzyme A desaturase 1 (SCD1), and fatty acid synthase (FAS). Furthermore, preclinical models have shown that berberine promotes fatty acid β‐oxidation via activation of Sirtuin 1 (SIRT1), contributing to its liver protective and lipid‐lowering actions [8]. Concurrently, berberine promotes the export of triglyceride and cholesterol from hepatocytes by upregulating microsomal triglyceride transfer protein (MTTP) and ATP‐binding cassette transporter A1 (ABCA1)—which mediates cholesterol efflux onto apoA‐I to form HDL particles [9].

Berberine also exhibits an anti‐inflammatory effect by suppressing pro‐inflammatory signaling (notably via inhibition of NF‐κB pathways) and lowers levels of cytokines and mediators such as IL‐6,IL‐8, IL‐1β, TNF‐α and COX‐2, which can attenuate liver inflammation and progression to steatohepatitis. Finally, berberine reduces oxidative stress by inducing antioxidant defense systems—upregulating enzymes and effectors including superoxide dismutase (SOD), the glutathione (GSH) system, and heme oxygenase‐1 (HO‐1), thereby decreasing lipid peroxidation and reactive oxygen species (ROS).

A recent meta‐analysis of 10 randomized control trials with 811 patients provided promising evidence that berberine, when used as an adjunct therapy, may confer additional benefits with minimal adverse effects for patients with MASLD, particularly in improving liver enzymes, dyslipidaemia, insulin resistance, and body weight [10]. Despite the possible improvements in metabolic markers and liver enzymes, the studies are small, follow‐up is short, and the between‐trial heterogeneity is substantial. Taken together, these data do not support a claim that berberine is a proven treatment for hepatic steatosis or fibrosis. Table 1 summarizes evidence evaluating Berberine in MASLD.

**TABLE 1:** Clinical evidence evaluating Berberine in metabolic dysfunction‐associated steatotic liver disease.

Study	Design	Population	Follow‐up	Primary outcomes assessed	Effect direction	Certainty of evidence	Key limitations
Nie et al. [10]	Systematic review and meta‐analysis (10 RCTs)	811 patients with MASLD/NAFLD	8–24 weeks (predominantly short‐term)	Liver enzymes, insulin resistance, lipids, body weight	Favorable (modest improvements in ALT, AST, HOMA‐IR, lipids, weight)	Low‐moderate	Heterogeneous interventions, variable trial quality, short duration, surrogate endpoints
Ilyas et al. [3]	Systematic review and meta‐analysis	12 studies in NAFLD/MASLD and metabolic disease populations	Variable	Metabolic and hepatic surrogate outcomes	Favorable but inconsistent	Low	High heterogeneity, limited liver‐specific endpoints, reliance on surrogate outcomes
Perez‐Rubio et al. [2]	Randomized placebo‐controlled trial	Adults with obesity and MASLD	24 weeks	Hepatic fat content, metabolic parameters	Neutral for hepatic fat; modest metabolic improvement	Moderate (single RCT)	Single‐center study, limited sample size, underpowered for liver endpoints, no histology
Yin et al. [11]	Randomized controlled trial	Type 2 diabetes/metabolic disease (~100–200)	~12 weeks	Glycaemic and lipid outcomes	Favorable metabolic effects	Low	Not MASLD‐specific, short duration, surrogate endpoints only
Zhang et al. [12]	Randomized controlled trial	NAFLD patients (~60–120)	12–16 weeks	ALT, surrogate steatosis markers, insulin resistance	Favorable	Low	Small sample size, no histological or imaging endpoints, short follow‐up
Wei et al. [13]	Randomized controlled trial	NAFLD patients (~80)	~16 weeks	Liver enzymes, metabolic indices	Favorable	Low	No histological endpoints, limited generalisability
Zhao et al. [14]	Randomized controlled trial	NAFLD patients (~90)	~12 weeks	ALT, triglycerides, HOMA‐IR	Favorable	Low	Short duration, heterogeneous reporting quality
Wang et al. [15]	Randomized controlled trial	NAFLD patients (~100)	8–24 weeks	Steatosis markers, metabolic parameters	Favorable but modest	Low	Small sample size, intervention heterogeneity, short follow‐up

Lifestyle modification remains the first‐line therapy for MASLD and can meaningfully improve hepatic steatosis when sufficient weight loss is achieved. Comparative evidence further underscores this hierarchy: in the network meta‐analysis by Wang et al., GLP‐1 receptor agonists produced the largest reductions in liver fat and steatosis, while PPAR‐based therapies ranked highest for BMI reduction [16]. Against this therapeutic backdrop, berberine's effects—typically modest reductions in body weight, improvements in insulin resistance, and decreases in liver enzymes—are comparatively limited, with little evidence to date for histological improvement or fibrosis regression. Accordingly, berberine is best positioned as a lower‐intensity adjunct rather than an equivalent alternative to established lifestyle interventions or evidence‐based pharmacologic therapies.

Berberine has poor systemic availability following oral administration due to extensive first‐pass metabolism and P‐glycoprotein‐mediated efflux; consequently, a divided daily dose of 1.0–1.5 g is standard practice in clinical studies. Across metabolic trials, berberine's measurable effects are usually observed over 8–12 weeks of therapy. Berberine is generally safe and mostly well tolerated, but adverse effects can occur. The most frequently reported side effects are predominantly gastrointestinal including: diarrhea, constipation, abdominal cramping, dyspepsia, and nausea. Transient headaches and a metallic taste have also been reported. Clinically significant drug–drug interactions may occur because of berberine's inhibition of CYP3A4, CYP2D6, and P‐glycoprotein, demanding caution when co‐administered with medications such as tacrolimus, cyclosporine, statins, anticoagulants, and other agents.

Further large‐scale, long‐term high‐quality multi‐center randomized controlled trials in MASLD that employ histological endpoints such as improvements in steatosis and fibrosis are needed. In addition to long‐term studies assessing both safety and sustained efficacy: head‐to‐head comparisons with established metabolic therapies, including GLP‐1 receptor agonists, pioglitazone, and metformin; mechanistic substudies examining pathways such as microbiome alterations and bile‐acid signaling; and formulation‐focused work aimed at enhancing its poor bioavailability. Moreover, future trials should prespecify safety endpoints and drug‐interaction monitoring, given berberine's documented effect on cytochrome P450 isoenzymes and P‐glycoprotein transport pathways. This is particularly pertinent when berberine is co‐administered with widely used cardiometabolic medications.

The designation “Nature's Ozempic” has contributed to berberine's prominence on social‐media platforms; however, it is not clinically rigorous or evidence‐based comparison. Semaglutide and other GLP‐1 receptor agonists have demonstrable, large effects on body weight and liver‐related outcomes, whereas berberine's effects are much smaller and less consistent. Figure 1 illustrates the contrast between the public perception of berberine as “Nature's Ozempic” and the current clinical evidence supporting its use. At this stage, clinicians should guide patients toward evidence‐based expectations and consider berberine cautiously, only as an adjunct therapy for selected metabolic conditions and not a “miraculous” weight loss drug inflated by social media claims.

**FIGURE 1:**
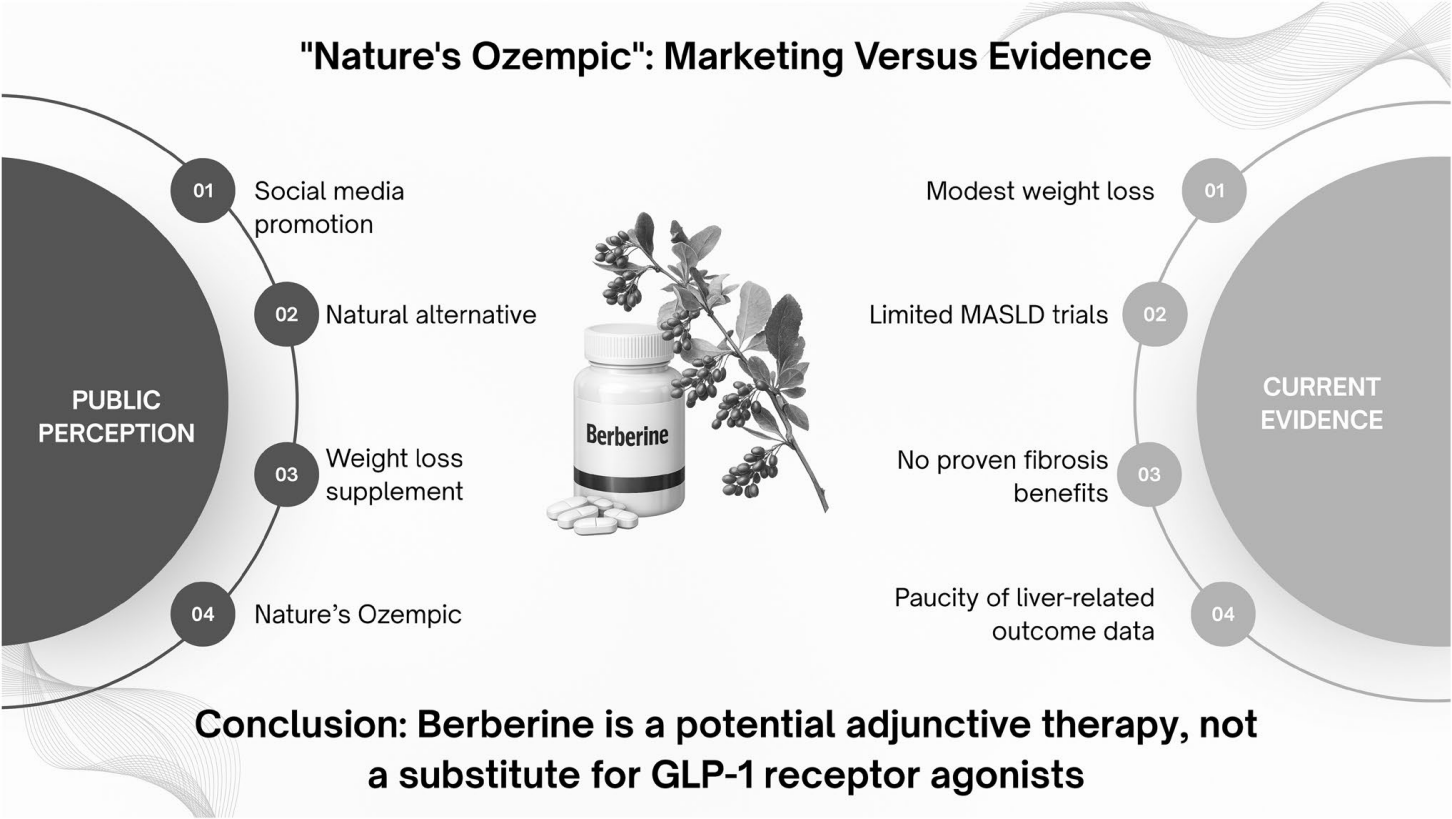
The “Nature's Ozempic” label for Berberine: Marketing versus evidence.

## Author Contributions

**Eric Kalo:** visualization, conceptualization, writing – review and editing, writing – original draft, investigation, methodology, formal analysis. **Ruth Nona:** writing – review and editing, visualization.

## Funding

The authors have nothing to report.

## Disclosure

This manuscript is a commentary and does not report new experimental or participant‐level data. The authors had complete access to the published sources cited in the manuscript and take responsibility for the accuracy of the cited information. There was no ongoing dataset accessed for the preparation of this commentary.

## Ethics Statement

The authors have nothing to report.

## Conflicts of Interest

The authors declare no conflicts of interest.

## Data Availability

Data sharing not applicable to this article as no datasets were generated or analysed during the current study.
